# Supplementary material for: Time-series transcriptome analysis identified differentially expressed genes in broiler chicken infected with mixed Eimeria species
Source: Front Genet. 2022 Aug 8;13:886781. doi: 10.3389/fgene.2022.886781 (PMC9393255; doi:10.3389/fgene.2022.886781)
Supplement: Supplementary file 2 [file DataSheet1.ZIP › 4dpi_KEGG.Gsea.1625100298584/KEGG_APOPTOSIS.html]

Details for gene set KEGG\_APOPTOSIS[GSEA]

|  || Dataset | TMM\_4dpi\_gct\_format\_4dpi\_gct\_format.Class\_4dpi.cls #PC\_versus\_NC.Class\_4dpi.cls #PC\_versus\_NC\_repos |
| Phenotype | Class\_4dpi.cls#PC\_versus\_NC\_repos |
| Upregulated in class | 1 |
| GeneSet | KEGG\_APOPTOSIS |
| Enrichment Score (ES) | 0.42781603 |
| Normalized Enrichment Score (NES) | 1.5500549 |
| Nominal p-value | 0.011853803 |
| FDR q-value | 0.2719957 |
| FWER p-Value | 0.9098 |
Table: GSEA Results Summary

  

Fig 1: Enrichment plot: KEGG\_APOPTOSIS      
 Profile of the Running ES Score & Positions of GeneSet Members on the Rank Ordered List

  

| SYMBOL | TITLE | RANK IN GENE LIST | RANK METRIC SCORE | RUNNING ES | CORE ENRICHMENT || 1 | CASP7 | na | 98 | 1.647 | 0.0561 | Yes |
| 2 | TNFSF10 | na | 174 | 1.398 | 0.1045 | Yes |
| 3 | FAS | na | 192 | 1.367 | 0.1565 | Yes |
| 4 | APAF1 | na | 235 | 1.286 | 0.2032 | Yes |
| 5 | MYD88 | na | 304 | 1.174 | 0.2434 | Yes |
| 6 | BIRC3 | na | 482 | 0.983 | 0.2669 | Yes |
| 7 | PIK3CA | na | 515 | 0.948 | 0.3013 | Yes |
| 8 | TRADD | na | 711 | 0.810 | 0.3166 | Yes |
| 9 | ENDOG | na | 742 | 0.791 | 0.3450 | Yes |
| 10 | FASLG | na | 769 | 0.773 | 0.3730 | Yes |
| 11 | PRKAR2B | na | 791 | 0.764 | 0.4011 | Yes |
| 12 | PRKAR2A | na | 925 | 0.700 | 0.4174 | Yes |
| 13 | CASP6 | na | 1314 | 0.572 | 0.4072 | Yes |
| 14 | CASP3 | na | 1333 | 0.565 | 0.4278 | Yes |
| 15 | CAPN1 | na | 1747 | 0.466 | 0.4114 | No |
| 16 | CAPN2 | na | 1917 | 0.437 | 0.4144 | No |
| 17 | CASP8 | na | 2138 | 0.398 | 0.4115 | No |
| 18 | PRKX | na | 2262 | 0.380 | 0.4160 | No |
| 19 | AKT3 | na | 2430 | 0.357 | 0.4160 | No |
| 20 | ATM | na | 2597 | 0.336 | 0.4152 | No |
| 21 | PIK3CB | na | 2649 | 0.329 | 0.4238 | No |
| 22 | FADD | na | 2797 | 0.309 | 0.4236 | No |
| 23 | ENDOD1 | na | 3142 | 0.263 | 0.4051 | No |
| 24 | CFLAR | na | 3205 | 0.256 | 0.4099 | No |
| 25 | DFFB | na | 3248 | 0.250 | 0.4162 | No |
| 26 | IKBKB | na | 3506 | 0.220 | 0.4032 | No |
| 27 | PRKAR1B | na | 3650 | 0.202 | 0.3992 | No |
| 28 | RIPK1 | na | 4121 | 0.158 | 0.3660 | No |
| 29 | TRAF2 | na | 4731 | 0.103 | 0.3191 | No |
| 30 | IL1RAP | na | 4854 | 0.093 | 0.3125 | No |
| 31 | CYCS | na | 5018 | 0.077 | 0.3019 | No |
| 32 | CASP9 | na | 5054 | 0.074 | 0.3018 | No |
| 33 | IRAK4 | na | 5151 | 0.064 | 0.2963 | No |
| 34 | AKT1 | na | 5330 | 0.052 | 0.2834 | No |
| 35 | PRKAR1A | na | 5407 | 0.045 | 0.2788 | No |
| 36 | BCL2L1 | na | 5617 | 0.024 | 0.2623 | No |
| 37 | AIFM1 | na | 5646 | 0.021 | 0.2608 | No |
| 38 | NFKBIA | na | 5648 | 0.021 | 0.2615 | No |
| 39 | RELA | na | 5979 | -0.005 | 0.2341 | No |
| 40 | PIK3R2 | na | 5995 | -0.006 | 0.2330 | No |
| 41 | BID | na | 6470 | -0.044 | 0.1951 | No |
| 42 | IL1R1 | na | 6480 | -0.045 | 0.1961 | No |
| 43 | PIK3R1 | na | 6590 | -0.054 | 0.1891 | No |
| 44 | NFKB1 | na | 6765 | -0.068 | 0.1771 | No |
| 45 | CHP1 | na | 6932 | -0.083 | 0.1665 | No |
| 46 | PRKACB | na | 7013 | -0.090 | 0.1633 | No |
| 47 | PPP3CA | na | 7298 | -0.117 | 0.1441 | No |
| 48 | TNFRSF1A | na | 7639 | -0.147 | 0.1214 | No |
| 49 | PPP3CB | na | 8043 | -0.184 | 0.0948 | No |
| 50 | IRAK2 | na | 8067 | -0.186 | 0.1002 | No |
| 51 | PIK3CG | na | 8485 | -0.227 | 0.0742 | No |
| 52 | PPP3R1 | na | 8746 | -0.255 | 0.0623 | No |
| 53 | DFFA | na | 8963 | -0.280 | 0.0552 | No |
| 54 | TNFRSF10B | na | 9083 | -0.293 | 0.0567 | No |
| 55 | PIK3R5 | na | 9216 | -0.311 | 0.0578 | No |
| 56 | CHUK | na | 9400 | -0.336 | 0.0556 | No |
| 57 | MAP3K14 | na | 9607 | -0.363 | 0.0525 | No |
| 58 | PIK3CD | na | 10148 | -0.437 | 0.0244 | No |
| 59 | CSF2RB | na | 10466 | -0.491 | 0.0170 | No |
| 60 | BCL2 | na | 10609 | -0.520 | 0.0255 | No |
| 61 | IL1B | na | 11033 | -0.617 | 0.0142 | No |
| 62 | EXOG | na | 11475 | -0.789 | 0.0081 | No |
| 63 | PIK3R3 | na | 11670 | -0.936 | 0.0285 | No |
Table: GSEA details [plain text format]

  

Fig 2: KEGG\_APOPTOSIS      
 Blue-Pink O' Gram in the Space of the Analyzed GeneSet

  

Fig 3: KEGG\_APOPTOSIS: Random ES distribution      
 Gene set null distribution of ES for **KEGG\_APOPTOSIS**
